# Determinants and aetiologies of postpartum pyrexia; a retrospective analysis in a tertiary health facility in the Littoral Region of Cameroon

**DOI:** 10.1186/s12884-020-02867-2

**Published:** 2020-03-17

**Authors:** Yannick Lechedem Ngunyi, Gregory Halle-Ekane, Nicholas Tendongfor, Etheldreda Leinyuy Mbivnjo, Armel Evouna Mbarga, Derick Nembulefack, Clifford Abonge Lo-oh, Thomas Obinchemti Egbe

**Affiliations:** 1 Faculty of Health Sciences, University of Buea, Buea, Cameroon; 2 Bebetta Memorial Community Clinic, Buea, Cameroon; 3 Obstetrics and Gynaecology Service, Douala General Hospital, Douala, Cameroon; 4 Biaka University Institute of Buea, Buea, Cameroon; 5 Cameroon Field Epidemiology Training Programme/Ministry of Public Health, Yaounde, Cameroon

**Keywords:** Postpartum pyrexia, Prevalence, Risk factors, Aetiologies, Douala general hospital, Cameroon

## Abstract

**Background:**

Postpartum febrile morbidity is relatively common, occurring in approximately 5–7% of births. Differentiating between potentially serious and benign causes of postpartum pyrexia (PP) is fundamental in curbing the mortality rate from sinister causes such as sepsis. The paucity of data on PP in Cameroon makes it difficult to access its actual burden. This study was aimed at determining the prevalence, risk factors and aetiologies of PP at a tertiary hospital in Douala, Cameroon.

**Methods:**

This was a 2 – year hospital – based retrospective cohort study carried out at the Douala General Hospital (DGH), during which medical records of all postpartum admissions between January 1st 2017 and December 31st 2018 were reviewed. The review consisted of collecting data on socio-demographic characteristics, clinical profile, investigations and final diagnoses. The collected data was analysed in SPSS 23.0. Chi-squared test was used to test the association between variables and a logistic regression analysis was fitted to identify risk factors associated to PP.

**Results:**

A total of 1520 postpartum files were reviewed. The prevalence of PP was 8.82%. The most frequent causes of PP were: malaria (46.7%), urinary tract infections (18.7%), puerperal sepsis (17.9%) and pneumonia (8.7%). *E. coli* was the most (49.3%) cultured germ isolated in positive cultures. Onset of PP was more common (85%) within the first 3 days postpartum and malaria (60%) was the leading aetiology within this period. Five or more vaginal examinations prior to delivery (OR 59.151, 95% CI: 21.463–163.019; *p* < 0.001), perineal tears (OR 45.157, 95% CI: 2.266–899.722; p < 0.001), and duration of labour > 18 h (OR 26.760, 95% CI: 7.100–100.862; p < 0.001) were the most significant risk factors associated with PP.

**Conclusion:**

Approximately 1 in every 12 postpartum cases in the DGH presents with PP**.** Malaria was the leading cause of PP at DGH especially for cases registered within 3 days postpartum. The risk factors identified were mostly associated to perinatal events, such as frequent vaginal examinations, perineal tears and prolonged labour. Efforts towards preventing identified risk factors thus becomes paramount in order to curb this high rate of PP in the DGH.

## Background

Postpartum pyrexia is any temperature rise above 38 °C maintained over 24 h or recurring between the end of the first to the end of the 10th day after childbirth [1]. Alternatively, the United States Joint Commission on Maternal Welfare defines postpartum pyrexia as temperature greater than 38.0 °C on any 2 of the first 10 days postpartum, exclusive of the first 24 h [1]. Single-day benign fever limited within the first 24 h postpartum is excluded because, it is very common and often resolves spontaneously, and cannot be explained by an identifiable postpartum aetiology [2]. Postpartum pyrexia is relatively common, occurring in approximately 5–7% of births [3], with global rates decreasing as progress is being made towards Sustainable Development Goal-3 [4]. However, postpartum pyrexia remains a leading contributor to maternal morbidity and mortality, especially in Sub-Saharan Africa, where 66% of maternal deaths occur [1]. Estimates of postpartum pyrexia rates in Africa are inconsistent, with rates higher than 64% observed in a Nigerian study in 2012 [5]. The lowest rate (2.9%) in Sub-Saharan Africa, was reported by a Ugandan study in 2018 [6]. In Cameroon there are no existing estimates of the prevalence of postpartum pyrexia. Studies in Yaoundé and Maroua Central Hospitals, reported that puerperal sepsis (the leading cause of postpartum pyrexia) accounts for approximately 4.2 and 14.3% of all maternal deaths respectively. Although there are various causes of fever during the postpartum period, infection is the most common cause, notably puerperal sepsis, which is ranked by the World Health Organization as the 6th leading cause of disease burden for women aged 15–44 years [4]. Rising rates of puerperal sepsis, has been strongly associated with nosocomial infections and increasing antibiotics resistance, posing a significant challenge to overburdened health care systems [4]. Previous studies reported that puerperal sepsis is a highly preventable problem occurring among the leading causes of maternal morbidity and mortality, not only in low income countries but also in high income countries as well [4, 5]. A study in Australia [7] identified the major causative microorganisms to be poly microbial with group A b-hemolytic *streptococcus*, often being the cause of severe cases of puerperal sepsis. Other significant causes of postpartum pyrexia include urinary tract infections, surgical wound infection, malaria, septic thrombophlebitis and mastitis [8]. In addition to trauma sustained during the birth process or caesarean procedure, physiologic changes during pregnancy contribute to the development of postpartum infections [3]. Other factors that increase susceptibility to sepsis and subsequent pyrexia in the postpartum period include anaemia, prolonged labour, frequent vaginal examinations in labour under unsterilized circumstances, and prolonged premature rupture of membranes [3, 9, 10]. Researchers have noted several risk factors associated postpartum pyrexia [10]. However, the relative importance of these risk factors varies with setting. Though an important maternal health issue in our setting, to the best of the authors’ knowledge, no study has been done in Cameroon to assess the disease burden posed directly by postpartum pyrexia; thus, there is lack of proper assessment of its impact on maternal morbidity and mortality. The rationale of this study was therefore to determine the prevalence, risk factors, and aetiologies of PP in our setting, so as to take necessary action towards its prevention and management.

## Methods

### Study design and setting

This was a hospital based retrospective cohort study, conducted between February and May 2019 in Douala general hospital (DGH), Cameroon. DGH is a tertiary hospital with four hospitalization units (medicine, surgery, pediatrics, and obstetrics), and well equipped laboratory and radiology units with highly trained staff. It has a well-organized gynecological/obstetric unit with obstetricians and gynecologists who provide all facets of perinatal care. It also has a well-equipped and functioning theatre that handles all obstetric surgeries. The hospital functions as a parastatal and is found in the city of Douala, Littoral region of Cameroon. Douala is the largest city in Cameroon with a population of more than 3.5 million inhabitants [11].

### Study population and sampling

The study population was made up of postpartum women admitted at the DGH between January 1st 2017 and 31st December 2018. Medical records of women in postpartum follow-up (women who gave birth at the DGH and women who were referred to the DGH for postpartum care following delivery in different hospitals irrespective of what they presented with) admitted in the DGH between 1st January 2017 and 31st December 2018 were analyzed. Incomplete files were excluded.

### Data collection

Data was collected using a predesigned data collection sheet. The women were placed in a cohort, those with postpartum pyrexia and those without. Data collected included; socio-demographic characteristics, clinical profile, investigations and final diagnoses or causes of PP.

### Statistical analysis

The collected data was entered into Microsoft excel and exported to SPSS version 24.0 for analysis. Chi-squared test was used to establish association between variables and risk factors were identified using a logistic regression analysis. The predictor variables were grouped into sociodemographic or obstetric. A *P*-value < 0.05 was considered significant.

### Ethics considerations

The ethical clearance for this study was obtained from the institutional Review Board of the Faculty of Health Sciences, University of Buea (ref. N^o^ 2019/887–01/UB/SG/IRB/FHS). An administrative approval was obtained from the Directorate of the Douala General Hospital, Cameroon (N^o^ 090 AR/MINSANTE/HGD/DM/O2/19). To ensure confidentiality, all patient information was coded.

## Results

During the study period, 1553 files were reviewed. Thirty-three files were incomplete, so were excluded, giving a total of 1520 complete files which were included. Of the 1520 records included in the study, 1502 (98.8%) gave birth at the DGH, and 18 (1.2%) women were referred for postpartum care at the DGH following delivery in different hospitals.

### Socio-demographic characteristics of the study population

Table 1 shows the sociodemographic and obstetric characteristics of participants. The subjects age ranged from 20 to 41 with mean age of 29.94 ± 4.9 years and majority, 1262 (83.0%) of the subjects were between the ages of 20–34 years.

**Table 1:** Sociodemographic characteristics of Study Population

Variable	Frequency	Percentage (%)
**Maternal age**		
20–34 years	1262	83
≥ 35	258	17.0
**Marital status**		
Single	441	29.0
Married	1079	71.0
**Education**		
Primary (primary school only)	145	9.5
Secondary (at most secondary and/or high school)	411	27.1
Tertiary (at least undergraduate level)	964	63.4
**Parity**		
Primipara	238	15.7
Multipara (2 to 4 births)	1010	66.5
Grand Multipara (5 or more births)	272	17.9

### Prevalence of postpartum pyrexia

Of the1520 files reviewed, 134 were cases of postpartum pyrexia, 88 out of 1018 vaginal delivery and 46 of the 502 caesarean section. This gave an overall postpartum pyrexia prevalence of 8.82%, with rates of 9.16% following caesarean section and 8.64% following vaginal delivery.

### Risk factors of postpartum pyrexia

The risk factors associated with PP were: five or more vaginal examinations prior to delivery (OR 59.151, 95% CI: 21.463–163.019; *p* < 0.001), perineal tears (OR 45.157, 95% CI: 2.266–899.722; *p* < 0.001), duration of labour greater than 18 h (OR 26.760, 95% CI: 7.100–100.862; p < 0.001), hemoglobin level <  8 g/dl prior to delivery (OR 23.960, 95% CI: 9.081–63.218; p < 0.001), pre-eclampsia (OR 5.710, 95% CI: 2.401–13.583; *p* = 0.040), grand multiparty (OR 3.878, 95% CI: 1.149–13.120; *p* = 0.029),less than 4 antenatal visits (OR 2.379, 95% CI: 1.321–6.975*; p* < 0.000) and caesarean delivery (OR 1.142, 95% CI: 0.000–8977.799; p < 0.001) (Table 2).

**Table 2:** Risk factors of postpartum pyrexia (Multivariate analysis)

Factors			***P***-value	AOR	Confidence Interval (95%)
**Parity**	**Primipara**		0.131	1.683	0.856–3.311
	**Multipara**			1	
	**Grand multipara**		**0.029**	3.878	1.149–13.120
**Comorbidity**	**HIV**	**Yes**	0.135	2.350	0.986–4.657
		**No**		1	
	**Diabetes**	**Yes**	0.356	1.678	0.564–2.463
		**No**		1	
	**Pre-eclampsia**	**Yes**	**0.001**	5.710	2.401–13.583
		**No**		1	
**Number of ANCs**	**< 4**		**0.001**	2.379	1.321–6.975
	**4–8**		0.067	0.079	0.213–1.875
	**> 8**			1	
**Hemoglobin level**	**< 8 g/dl**		**0.001**	23.960	9.081–63.218
	**8.1–10 g/dl**		0.997	0.073	0.033–0.161
	**> 10 g/dl**			1	
**Duration of labour**	**< 18 h**			1	
	**> 18 h**		**0.001**	26.760	7.100–100.862
**Number of VEs**	**< 5**			1	
	**5 and above**		**0.001**	59.151	21.463–163.019
**Mode of delivery**	**Vaginal**			1	
	**Instrumental**		0.977	0.210	0.098–0.448
	**Caesarean section**		**0.001**	1.142	0.001–8977.799
**Tears**	**Yes**		**0.013**	45.157	2.266–899.722
	**No**			1	

*AOR* Adjusted odd ratios, *VE* vaginal examination, *ANC* antenatal clinic

### Etiologies of postpartum pyrexia in Douala general hospital

**Fig. 1:**
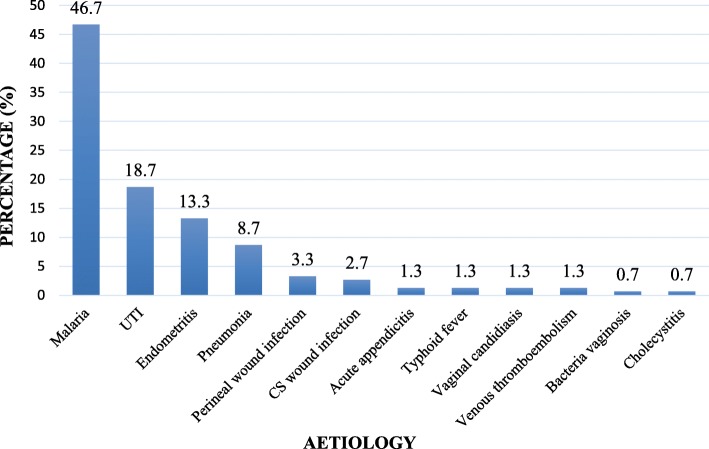
Aetiologies of postpartum pyrexia in Douala general hospital

Malaria was the most common cause of postpartum pyrexia 70 (46.7%). Other causes identified included urinary tract infections, 28 (18.7%), endometritis, 20 (13.3%), pneumonia, 13 (8.7%), perineal wound infection, 5 (3.3%), CS wound infection, 4 (2.7%), acute appendicitis, 2(1.3%), typhoid fever, 2 (1.3%), vaginal candidiasis, 2 (1.3%), venous thromboembolism (1.3%), bacterial vaginosis, 1 (0.7%) and cholecystitis, 1 (0.7%). 12 (9.0%) of women had both malaria and UTI, 3 (2.2%) had UTI and endometritis and 2 (1.4%) had endometritis and perineal wound infection. (Fig. 1).

Approximately 73.3% of all cases of postpartum pyrexia started within days 1 to 3 postpartum. Malaria 60.0% (66/110) was the leading cause of fever within the first 3 days postpartum. Between days 4 to 6, postpartum pyrexia was mainly caused by pneumonia 28.6% (6/21), UTI 23.8% (5/21), malaria 19.0% (4/21) and endometritis 9.5% (2/21). From day 7 and above, the main cause of postpartum pyrexia was endometritis 72.2% (13/18), perineal wound infection 16.7% (3/18) and venous thromboembolism 11.1% (2/18) (Table 3).

**Table 3:** Aetiologies of PP in relation to day of onset postpartum, DGH 2017–2018, Cameroon

Causes	Day of Onset of Postpartum		
	Day 1 to 3	Day 4 to 6	Day 7 to 10
Malaria	66	4	0
UTI (pyelonephritis)	23	5	0
Pneumonia	7	6	0
Endometritis	5	2	13
Acute appendicitis	2	0	0
CS wound infection	2	2	0
Typhoid fever	2	0	0
Vaginal candidiasis	2	0	0
Bacterial vaginosis	1	0	0
Perineal wound infection	0	2	3
Venous thromboembolism	0	0	2
**TOTAL**	**110**	**21**	**18**

### Microbiological profile of causative agents of postpartum pyrexia

For infectious causes, 89 culture results were reviewed, 20 of which were sterile. The most commonly cultured specimen were urine and high vaginal swabs. *E. coli* (51.5%) was the most isolated organism (Tables 4 and 5). Other microorganisms isolated were *Klebsiella pneumoniae, Trichomonas vaginalis, Streptococcus pyogenes, Staphylococcus aureus, Streptococcus pneumonia, Streptococcus agalactiae, Neisseria gonorrhoea, Ureaplasma urealyticum, Candida albicans and Salmonella typhi*.

**Table 4:** Isolated organisms of cultured specimens, DGH 2017–2018, Cameroon

Specimen	Frequency	Percentage	Isolated organisms
High vaginal swab	26	29.2	*E. coli, S.pyogenes, N.gonorrhoea,* *U.urealyticum, C.albicans,* *S.agalactiae, T. vaginalis*
Sputum	3	3.4	*S. pneumonia*
Urine	50	56.2	*Escherichia coli,* *Klebsiella pneumoniae*
Wound swab	7	7.8	*S. aureus*
Blood	3	3.4	*S.typhi* *S. pneumonia*
**Total**	**89**	**100.0**	10

**Table 5:** Results of patients with positive cultures, DGH 2017–2018, Cameroon

Organism	Number	Percentage
*Escherichia coli*	34	49.3
*Klebsiella pneumoniae*	6	8.7
*Trichomonas vaginalis*	6	8.7
*Streptococcus pyogenes*	5	7.2
*Staphylococcus aureus*	4	5.8
*Streptococcus pneumonia*	4	5.8
*Streptococcus agalactiae*	2	2.9
*Neisseria gonorrhoea*	2	2.9
*Ureaplasma urealyticum*	2	2.9
*Candida albicans*	2	2.9
*Salmonella typhi*	2	2.9
**TOTAL**	**69**	**100**

## Discussion

This study aimed to determine the prevalence, risk factors and aetiologies of postpartum pyrexia in the DGH. We found out that approximately 1 of every 12 women (8.82%) had postpartum pyrexia, slightly edging the global prevalence of 5–7% [3]. A similar study done in Ethiopia in 2014 [12], revealed a similar prevalence of 8.4%. The prevalence gotten in this study was far less, compared to that of a study carried out in Nigeria [5], which revealed a prevalence of 64.44%. The latter’s exceptionally high prevalence could have been because the study was limited to a small sample size consisting of already sick postpartum women, unlike in our study where all postpartum women were included. In a similar study carried out in Uganda in 2018 [6], the prevalence of postpartum pyrexia was 2.9%, much less compared to our study. Of note is the fact that wound infection and non-infectious causes of postpartum pyrexia were excluded in the Ugandan study, and this could explain the lower prevalence gotten compared to our study, which explored all aetiologies. A study in the USA [13] reviewed ambulatory medical records and relevant hospital records postpartum and computed an overall postpartum pyrexia incidence of 6.0%, with rates of 7.4% following caesarean section and 5.5% following vaginal delivery. Similar rates of 6.3% following caesarean section were gotten in another US study [14]. Also, large-scale post discharge surveillance in an American hospital following vaginal delivery and caesarean section, identified a 4% postpartum pyrexia rate [15]. These findings are lower compared to the ones gotten in this study; 9.16% following caesarean section and 8.64% following vaginal delivery. This may be explained by the fact that the American studies were done in a developed setting, where infectious diseases are less prevalent due to high standards of infection control. A majority of those with postpartum pyrexia were observed in the 20–34 year age group. However, with the exception of grand multiparity, no socio-demographic factor was found to be significantly associated with postpartum pyrexia. Risk factors identified included: five or more vaginal examinations prior to delivery, perineal tears, prolonged active phase of labour greater than 18 h, anemia prior to delivery, pre-eclampsia, grand multiparty, less than 4 antenatal visits and caesarean delivery (in order of decreasing significance). Most of these factors are in keeping with a review for low and middle income countries in 2012 [16]. Our findings also corroborate with those of a study carried out in Ethiopia [14] which reported caesarean delivery and anemia as significant risk factors of postpartum pyrexia. Also, studies in Nigeria [5, 9], revealed that frequent vaginal examinations was associated with significant risk of puerperal sepsis, and subsequent pyrexia. However, our findings contrary to other studies [3, 12], found no association between PP and prolonged rupture of membranes (PROM). This is probably due to routine use of broad spectrum antibiotics in patients with PROM at the DGH, as prophylaxis for chorioamnionitis and subsequent neonatal sepsis. In contrast to previous studies [17–19], there was no significant association between postpartum pyrexia and comorbidities like HIV infection, diabetes and obesity. These were large sample sized prospective cohort studies involving postpartum women, who were followed over long periods. Our study may have been limited because of the retrospective design and a short duration of study. Contrary to previous reviews [3, 10] which reported puerperal sepsis as the leading cause of postpartum pyrexia, malaria was the single most frequent cause of postpartum pyrexia observed in this study. The high incidence of postpartum malaria observed is typical to that described in a Gabonese study [20], which revealed that puerperal women were susceptible to a considerable risk of developing malaria. This finding is also in keeping with a similarly higher incidence of postpartum malaria reported in Nigeria [5]. This high incidence is probably due to the similarities in demographics between Cameroon, Nigeria and Gabon, were malaria is highly endemic, coupled with inconsistent or poor adherence to the intermittent preventive measures against malaria among pregnant women in these countries. Bacterial infection was the most prevalent etiology associated with postpartum pyrexia cumulatively. UTI (18.7%) and puerperal sepsis (17.9%) mostly in the form of endometritis, were the two most commonly identified infections in our study. This finding differs from that of an Ethiopian study, which noted a lower rate of UTI (14%) and very high rate of puerperal sepsis (39%). Given that onset of postpartum pyrexia due to puerperal sepsis is more common after the first week postpartum, which is usually post-discharge, most cases usually go undiagnosed. The retrospective design of our study hindered follow up of patients post discharge, so most of the late cases of postpartum pyrexia may have been missed, which was not the case with the above study which had a prospective design. This could explain their higher prevalence of puerperal sepsis. Approximately 77.5% of culture results reviewed in this study were positive, with the most isolated germ being *E. coli*. This finding is similar to those revealed in other studies [21, 22] which identified *E. coli* as the most commonly isolated organism from high vaginal swabs obtained from women with puerperal sepsis. However, these studies were limited to high vaginal swab cultures among women with puerperal sepsis and so may not reflect the true microbiological spectrum of postpartum pyrexia, compared to our study which reviewed culture results from different specimen. Six different species of bacteria were observed from high vaginal swabs cultures reviewed, with *E. coli* and group-A beta hemolytic streptococci being the most frequent. This result is highly reflective of the polymicrobial nature of puerperal sepsis, as reported in literature [3, 10, 21, 23]. The importance of timing the onset of pyrexia postpartum, cannot be over emphasized. It is an important indicator of the possible aetiology and management strategy to be implored by the care giver. We observed in our study that pyrexia was more common within first 3 days postpartum. This finding is contradictory to a previous review [13] in which 94% of cases of postpartum pyrexia, mostly secondary to postpartum infections occurred several days post discharge. This discrepancy can be explained by that fact that malaria was the single most common cause of postpartum pyrexia in our study, as opposed to bacterial infections which often present late, and are often diagnosed on readmission several days postpartum.

## Limitations of study

The Douala General Hospital is a tertiary hospital with one of the highest standards and cost of care in Cameroon. High cost of care may have increased the probability of getting patients only from a certain class in the society. Hence the prevalence of postpartum pyrexia gotten from this study, may not be very reflective of the actual burden in the country, as majority of people seek care only in primary care centers where there are usually sub-standard infection control measures, and probably a higher prevalence of postpartum pyrexia. We recommend further research on this subject using prospective study designs, in primary secondary health facilities.

## Conclusion

In the Douala General Hospital, the prevalence of PP was 8.82% with a rate of 1 in every 12 postpartum admissions. The risk factors were mainly perinatal factors such as frequent vaginal examinations during labour, perineal tears and prolonged labour. Postpartum pyrexia was more prevalent on the first 3 days postpartum, and was most commonly due to postpartum malaria. UTI and puerperal sepsis, in keeping with data from other studies in Africa, were also very significant causes of postpartum pyrexia in DGH, with *E. coli*, being the most implicated causative agent. The high rate of postpartum malaria noted in this study, is highly suggestive of poor adherence to preventive measures of malaria during pregnancy, such as the use of insecticide treated mosquito nets and intermittent preventive treatment of malaria. It is therefore fundamental that these women be sensitized during antenatal care on the importance of adhering to these measures, and also for the state to ensure that, these facilities are always available to these women free of cost. Also efforts towards preventing identified risk factors of PP thus become paramount in order to curb this high rate of postpartum pyrexia in our hospitals and community at large.

## Data Availability

The data sets supporting the findings of this study are available from the corresponding author on reasonable request.

## Abbreviations

AIDS: Acquired immune deficiency syndrome
ANC: Antenatal clinic
AOR: Adjusted odd ratio
CS: Caesarean section
DGH: Douala General Hospital
HIV: Human immunodeficiency virus
HVS: High vaginal swap
ICD: International classification of disease
PP: Postpartum pyrexia
PROM: Premature rupture of membranes
UTI: Urinary tract infection
VE: Vaginal examination
WHO: World Health Organization
